# A roadmap for managing an ageing workforce in the manufacturing sector: An Italian case study

**DOI:** 10.12688/openreseurope.20511.1

**Published:** 2025-08-26

**Authors:** Niloofar Katiraee, Giulia Caprari, Ajay Das, Daria Battini

**Affiliations:** 1 Universita degli Studi di Padova Dipartimento di Tecnica e Gestione dei Sistemi Industriali, Vicenza, 36100, Italy; 2 Baruch College Zicklin School of Business, New York, USA

**Keywords:** Ageing workforce management, knowledge transfer, worker retention, labour shortage, manufacturing system, ergonomic assessment, case study

## Abstract

**Background:**

Managing an aging workforce presents increasing challenges across industries, particularly in physically demanding sectors such as manufacturing. Key issues include knowledge transfer, worker retention, and maintaining productivity in operational environments that strain older workers. Northern Italy, a region with a strong manufacturing base, provides a relevant context for examining these challenges. This study applies the "Models and Methods for an Active Ageing Workforce" (MAIA) framework, which addresses six domains critical to supporting older workers: organizational culture, work design, health management, knowledge transfer, intergenerational coexistence and retirement pathway.

**Methods and Results:**

A case-based methodology was employed within a multinational manufacturing company in Northern Italy. Data were collected through qualitative and quantitative approaches, including stakeholder interviews, workplace observations, and targeted surveys assessing physical and mental workload. Analysis of this data, combined with discussions involving managers, foremen, and HR representatives, informed the development of a comprehensive roadmap for managing the aging workforce. The roadmap identifies key addressed areas and critical gaps and proposes targeted tools and actions to improve work conditions for older employees. This approach demonstrates the practical application of the MAIA framework and the ISO 25550:2022 standard in an industrial aging workforce context.

**Conclusions:**

The study highlights the importance of a structured framework for managing aging workers in physically demanding environments. By identifying specific challenges and proposing actionable solutions, the developed roadmap supports effective workforce planning and management. The results contribute valuable insights into optimizing organizational culture, health management, knowledge transfer, and retirement planning to promote sustainable employment for older workers in manufacturing.

## Plain language summary

As people work longer and populations age, many industries face challenges managing older workers, especially in physically demanding jobs like manufacturing. This study looks at a manufacturing company in Northern Italy to understand how to better support older employees. We used a framework called MAIA that focuses on six important areas, including workplace culture, job design, health, knowledge sharing, how different generations work together, and retirement planning.

We collected information by talking to workers and managers, observing the workplace, and using surveys to measure the physical and mental demands of the job. Based on this information, we developed a detailed roadmap that not only outlines ways to improve working conditions and support older workers but also highlights what actions have already been implemented and which key areas have been addressed within the company. This roadmap identifies gaps and suggests further actions and strategies that can be taken to better support the aging workforce. While we are still evaluating its full impact, we believe this roadmap can serve as a valuable guide to help companies plan and implement effective measures for an aging workforce.

## 1. Introduction

As populations age worldwide, the workforce is undergoing a major demographic shift, which is reshaping how societies, economies, and organizations function ( Zhao, 2024). Industrial sectors are experiencing a rise in the proportion of older workers, bringing both challenges and opportunities for companies ( Boenzi *et al.*, 2015). This is particularly evident in manufacturing roles, where the physical and cognitive demands are high, necessitating innovative strategies to ensure productivity while safeguarding worker well-being ( Goyal *et al.*, 2014).

Replacing aging workforces or early retirement is not always a feasible solution, as it can lead to the loss of valuable accumulated knowledge and expertise ( Streb *et al.*, 2008). Additionally, a shrinking pool of younger workers is creating labor shortages, leaving companies with little choice but to develop strategic approaches for managing an aging workforce ( Leibold & Voelpel, 2007). Thus, organizations must create age-friendly environments that prioritize health, enhance motivation, and ensure the engagement of older employees to maintain productivity and facilitate knowledge transfer to younger generations ( Katiraee *et al.*, 2024; Ranasinghe *et al.*, 2024). Intergenerational harmony is critical to the long-term success of companies, especially as they navigate this demographic shift.

The core challenge of effectively managing aging workforces lies in a) documenting and transferring their valuable experience and knowledge along with b) finding measures to decelerate and cope with the gradual decline in their physical and cognitive abilities. The ‘how’ of doing so constitutes the subject of the current study.

The objective of this study is to examine the complexities of knowledge transfer, worker retention, and productivity within an aging workforce in the manufacturing sector located in northern Italy.

An empirical study benefits from a sound conceptual point of departure. Studies have proposed conceptual frameworks for managing the aging workforce (i.e., Katiraee *et al.*, 2024; Rasmussen, 1997; Wilckens *et al.*, 2020). One such framework is the Models and Methods for an Active Ageing Workforce (MAIA) ( Figure 2), developed through international collaboration and data collection ( Katiraee *et al.*, 2024). While frameworks such as Rasmussen’s focus primarily on occupational safety and major accidents, and Wilckens *et al.*, 2020 address aging workforce challenges without considering multi-level interactions, the MAIA framework provides a more comprehensive approach. It accounts for new and evolving issues of aging workforces, such as ergonomics, health management, knowledge transfer, and retirement pathways, and does so across three levels—international, country, and company. Additionally, the MAIA framework was developed and refined through data collection and analysis from multiple project partners, incorporating insights from diverse regions and organizations. This iterative development process ensures the framework is tailored to address the current and emerging needs of aging workforces. While the framework provides valuable conceptual insights, its practical applicability and utility in real-world settings are still in an exploratory stage. Accordingly, we use the MAIA framework to operationalize and guide our empirical investigation.

We applied the MAIA framework in a large multinational manufacturing company that was developing guidelines for creating a more age-inclusive and sustainable work environment. We utilized the MAIA framework to structure and conduct a series of interviews with department heads, manual workers, and HR personnel to better understand and address the complexities of knowledge transfer, worker retention, and productivity in an operational environment with an aging workforce.

The following sections provide a theoretical background on the challenges of managing an aging workforce ( Section 2) and the methods to address them. Section 3 describes the research methodology, followed by observations and an in-depth analysis of the results in Section 4. Finally, the discussion and conclusion are presented in Section 5 and Section 6, respectively.

## 2. Theoretical background

### 2.1 Aging workforce challenges

**Figure 1.**
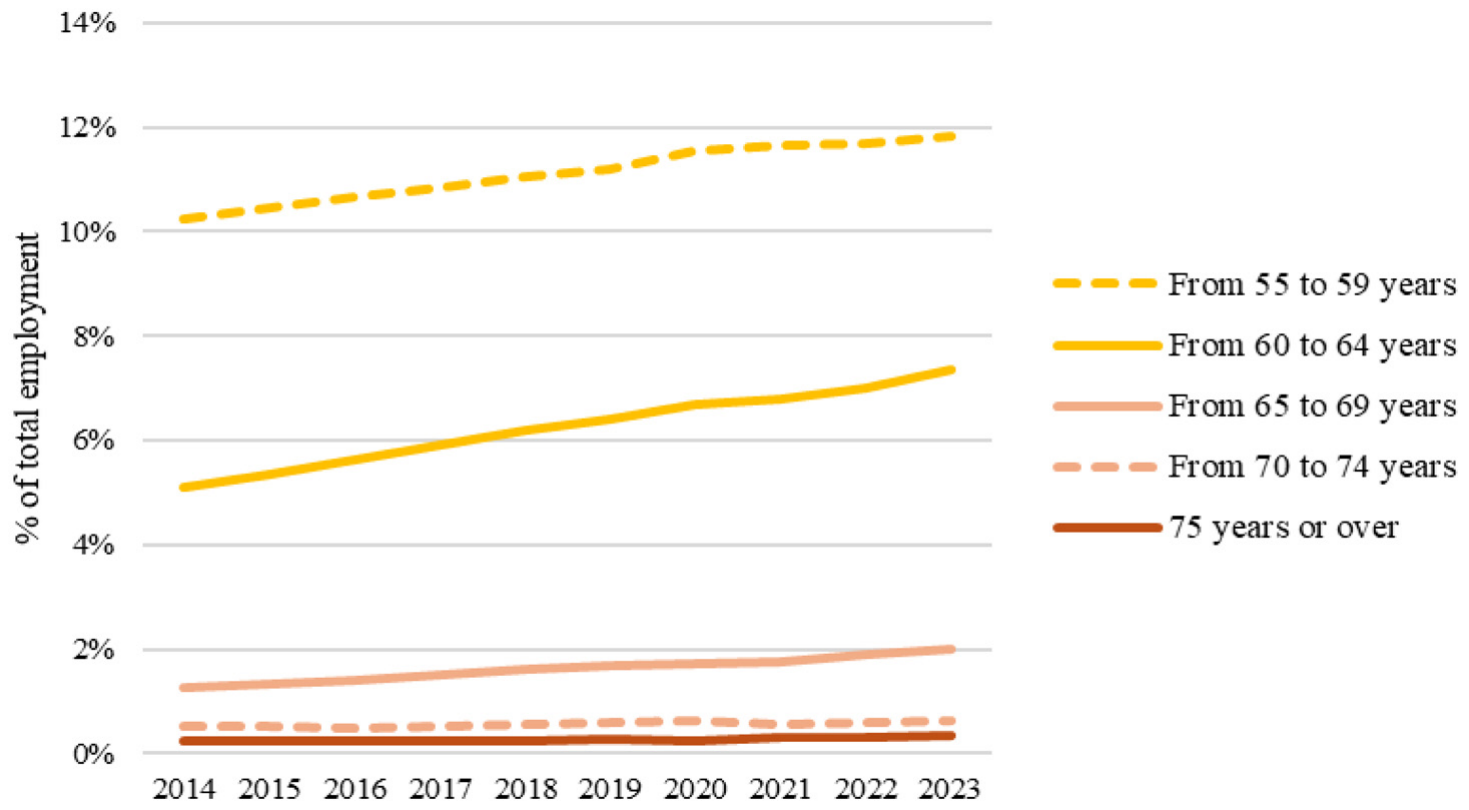
Older persons in employment, by age class, EU-27, 2014–2023 (% of total employment). Source: Eurostat ( lfsa_egan).

The world is experiencing a significant demographic shift, with the proportion of older workers growing rapidly ( Katiraee *et al.*, 2020). This change, driven by low fertility rates and increased life expectancy, is placing pressure on companies, especially in manufacturing systems, where physical demands are high, such as in manual assembly systems or tasks in warehouses. Figure 1 illustrates the upward trend in the proportion of aging employees across various age groups over the last decade.

The core challenge of aging workforces lies in balancing their experience and knowledge which have accumulated over time ( DeLong, 2004), with the decline in their physical and cognitive abilities ( Fisher *et al.*, 2017). These contrasting factors create difficulties for decision-makers in the workplace. Furthermore, integrating new technologies (e.g., cobots and digital tools) presents additional challenges. Older workers may face difficulties in adapting to new technologies, and the process of learning and training can be time-consuming and challenging compared to younger employees ( Sanders, 2018). Given these complexities, several important questions arise:

•   How can managers and organizations effectively address these challenges?

A common solution, as proposed in the literature, involves developing tailored strategies and guidelines specific to each company, depending on the organization’s requirements and the nature of the task being performed by employees.

•   Can these strategies be generalized for all workers? Should workers be treated equally?

Previous research, including studies by Katiraee *et al.* ( 2019; 2021a) and the recent ISO standard ISO 25550:2022, emphasizes the importance of individualization. Workers may differ significantly in terms of age, gender, skills, and physical or mental capabilities, suggesting that a one-size-fits-all approach may not be effective.

•   Should early retirement be considered as a solution?

While early retirement might seem like a straightforward solution, it often results in losing valuable knowledge and experience. Additionally, it can lead to significant economic costs related to retirement pensions and policies, which can impact both companies and governments.

For this reason, managing an aging workforce presents significant challenges for managers and practitioners. To address these issues, various frameworks and guidelines have been developed, including the European MAIA project ( Katiraee *et al.*, 2024). In the next subsection, we will provide a brief introduction to the MAIA framework.

### 2.2 Introduction to the MAIA framework

**Figure 2.**
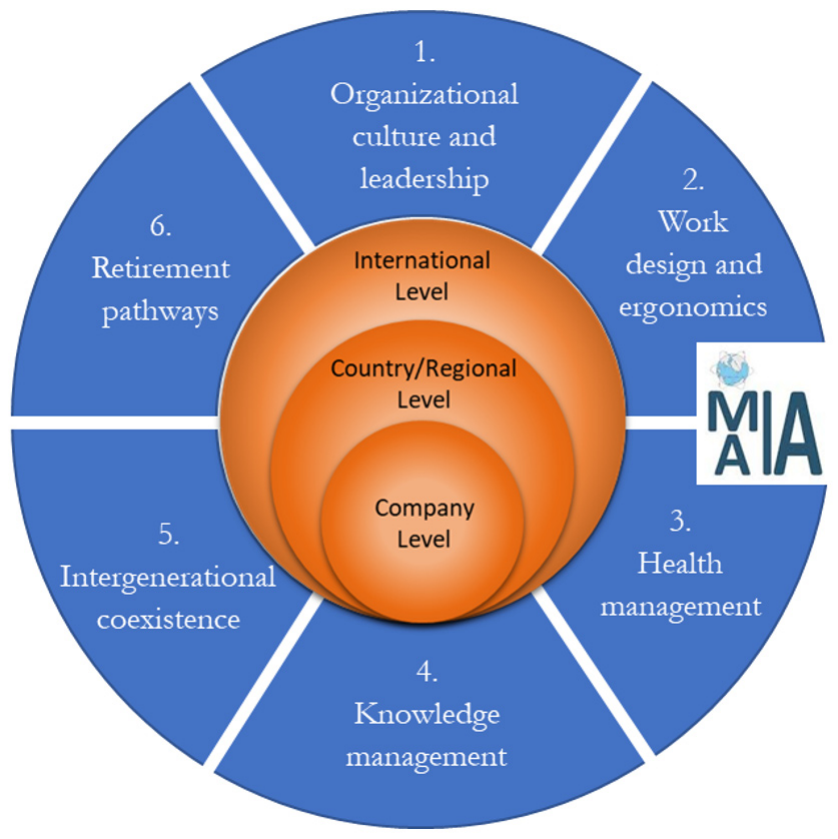
MAIA roadmap for implementing an age-inclusive workforce management practice in worldwide companies ( Katiraee *et al.*, 2024).

Managing an aging workforce is crucial for maintaining productivity, reducing health risks, and preventing knowledge loss ( DeLong, 2004). Strategies such as ergonomic workplace design, flexible work arrangements, and knowledge management systems are essential for fostering an age-inclusive environment ( Katiraee *et al.*, 2024; Ranasinghe *et al.*, 2024). The MAIA framework offers a structured approach to addressing these challenges across six key domains. Figure 2 illustrates the MAIA framework. A brief explanation of this framework is provided in this section, and further details can be found in Katiraee *et al.* (2024).

As shown in Figure 2, the MAIA framework offers a roadmap includes six main action domains and three levels of interventions. The domains are: 1) Organizational culture and leadership, 2) work design and ergonomic, 3) health management, 4) knowledge management, 5) intergenerational coexistence, and 6) retirement pathways. These domains are structured across three levels including international, country, and company level.

According to this roadmap, several key issues related to the aging workforce need to be addressed, including organizational culture, work design, worker’s health, knowledge transfer and communication among different groups of employees, and retirement planning. Policies, strategies, and guidelines applied for each domain have been discussed completely across all three levels (international, country, and company) by Katiraee *et al.*, 2024. The roadmap has been refined based on data gathered from worldwide partners involved in this project, ensuring a comprehensive and globally informed perspective.

We applied the MAIA roadmap in a real-world setting, a manufacturing company specializing in the production of agricultural machines / electronic devices, to identify the complexities of knowledge transfer, worker retention, and productivity in an aging operational environment. In the following section, we outline the company description and methodology used for data collection and analysis.

## 3. Methods

### 3.1 The company case/ case study introduction

The company under study is an Italian firm specializing in advanced industrial electronic components with over 1,500 employees globally, operating numerous production sites in Italy and abroad, sales offices, and local representative offices. The studied company is guided by core values, including sustainability, trust, people development, independence, partnership, and ambition. Since 2020, the company has adopted an "adaptive organization" model where Agile Methodologies such as Scrum and Kanban are integral to the company's approach, promoting collaboration, accountability, and continuous improvement.

The company is committed to creating a positive organizational climate, particularly for its aging workforce, with a specific focus on considering the physical limitations of the workers and improving knowledge transfer processes post-retirement. This study focuses on the Italian workforce, particularly employees aged 55 and above, who make up a significant portion of both blue-collar and white-collar roles. To achieve this, the company utilized the MAIA roadmap to gather data for analysis. The aim was to identify areas of the workplace environment that require further improvement, those that are already well-managed, and to outline actions that need to be initiated from scratch.

Table 1 provides an analysis of the aging workforce in the case studied across various roles for both blue- and white-collar workers, detailing the proportion of workers aged 55 or older, the average workforce age in each role, and early retirement preferences. The data includes the total workforce for both categories, encompassing both permanent and temporary employees. The inclusion of temporary workers reflects the company's practice of hiring seasonal staff during peak demand periods, highlighting the dynamic nature of its workforce. Notably, physically demanding blue-collar roles, such as assembly line and warehousing, have a significant proportion of aging workers, with approximately 90% indicating interest in early retirement based on a performed survey (detail in Section 4). The table also highlights the implications of these trends. For instance, in the warehousing role, the relatively high average age of 50.4 suggests that, within a decade, a considerable portion of the workforce may retire, assuming all workers retire at the standard age of 66–67, potentially leading to a workforce gap. If pre-retirement trends are factored in, this shortage could become even more severe, presenting challenges related to the loss of workforce knowledge, skills, and experience, and intensifying replacement issues.

**Table 1.** Workforce age distribution in the studied company for each role.

Collar	Role	Total workers (permanent and temporary)	Total workers aged 55 and above	%of the workforce aged 55 or more	Average workforce age	The expected request for early retirement
Blue Collar 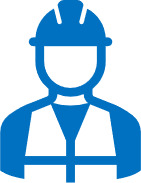	Assembly line	170	27	15.88%	46.1	
	Pick and place	53	13	24.53%	47.7	
	Warehouse	26	12	46.15%	50.4	
	**Total**	**249**	**52**	**20.88%**	**48.06**	**90%**
White collar 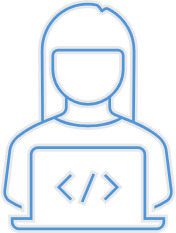	R&D	96	18	18.75%	43.7	
	Operations	139	17	12.23%	41.6	
	Marketing and sales	77	11	14.29%	42.1	
	Finance and Legal	30	4	13.33%	38.32	
	HR	31	3	9.68%	36.5	
	IT	15	2	13.33%	36.7	
	**Total**	**388**	**55**	**14.18**%	**39.8**	

In contrast, white-collar roles exhibit a lower proportion of aging workers and a younger average age overall. However, roles like R&D and operations still have a considerable number of workers aged 55 or older, underlining the need for strategic workforce planning across all levels of the organization.

To ensure operational excellence, the company identifies critical roles within the organization based on three criteria: unique expertise required, limited availability of skills in the labour market, and strategic importance to the company. Table 2 presents these roles, which were identified through interviews.

- Roles are currently held by workers aged over 55.
- Roles are filled by younger workers.
- Roles remain unfilled due to a lack of suitable replacements.

**Table 2.** Critical roles.

	Function	Role	Critical tasks
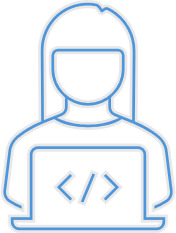	R&D	Plastic injection molding expert	How to design a piece in order to set up a feasible industrialization
	R&D	Electronic engineer	Electronic design and testing experience
	R&D	Hardware engineer	Hardware testing set up
	R&D	Mechanical designer	How to design a piece able to solve specific problems
	R&D	Patents expert	Document management about patents releases
	R&D	Product encoding expert	BOM knowledge about products
	R&D 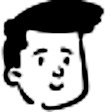	Fluid-dynamic simulations engineer	Virtual product testing
	Operations	Electronic process engineer	Electronic parts production processes
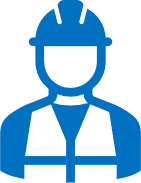	Operations	Press foreman	Sheet metal forming - deep drawing: codesign with the supplier to define the mold specifications and how to do the machines set up
	Operations	Plant manager	Management skills to manage workers in the factory
	Operations	Maintenance coordinator (unfilled)	Electrical and mechanical maintenance in production lines
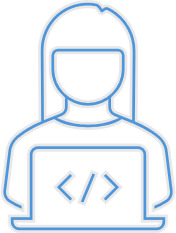	Marketing & Sales	Sales manager	Historical knowledge about product and customer (especially the human aspect)
	Marketing & Sales	Sales manager	Historical knowledge about product and customer (especially the human aspect)

Using the information about the company, we selected a representative sample for our survey and distributed the questionnaire, as outlined in Section 3.2.

### 3.2 The Interview sample and data analysis process

In this study, we adopted a case-based research approach ( Yin, 2009) and used unstructured and semi-structured interviews for data collection ( Podsakoff *et al.*, 2016). This methodology is particularly effective for capturing respondents' experiences and perceptions of performing tasks within the company.

Our focus was on employees aged 55 and older in a large multinational manufacturing company that was implementing age-inclusive guidelines. The sample comprised 103 interviews with employees from various departments. Data was gathered from two distinct groups of permanent workers: blue-collar and white-collar. Temporary workers were excluded from the survey due to limited access to information and constraints in conducting interviews.

Blue-collar workers were involved in manual and physically demanding tasks, such as assembly and warehouse operations, while white-collar workers were engaged in administrative tasks, such as R&D and IT. Table 3 presents a breakdown of the interview sample across both blue-collar and white-collar roles, along with their respective departments, providing a clearer understanding of the sample examined in this study.

**Table 3.** A breakdown of the interview sample across blue-collar and white-collar roles.

Collars (Blue & White)	Total permanent workers	Total workers aged 55 and above	Roles #Number of 55+ workers
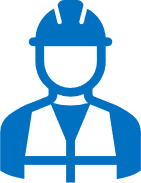	198	52	• Assembly line (#27) • Pick and place (#13) • Warehouse (#12)
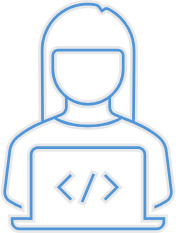	385	55	• R&D (#18) • Operations (#17) • Marketing and sales (#11) • Finance and legal (#4) • HR (#3) • IT(# 2)

The interview questions were designed in alignment with the six domains of the MAIA framework, tailored to address the research aim, and customized for both blue-collar and white-collar workers. For certain domains, such as Domain 2: Work Design and Ergonomics, the questions were developed using the simplified NASA-TLX to assess mental and physical workload ( Noyes & Bruneau, 2007). Additionally, to validate the qualitative data for this domain, quantitative ergonomic tools like RULA (Rapid Upper Limb Assessment) ( McAtamney & Corlett, 1993) and REBA (Rapid Entire Body Assessment) ( Hignett & McAtamney, 2000) were used to measure the physical strain experienced during critical tasks. For other domains, particularly Domain 1 (organizational culture and leadership) and Domain 6 (retirement pathway), the open-ended questions were crafted to reflect the specific characteristics and operational needs of the company. These questions were designed to be flexible, allowing the interview to naturally expand into more detailed discussions, enabling deeper insights into the issues at hand. Domain 5, concerning 'Intergenerational Coexistence,' was not considered in the analysis due to the difficulty in obtaining the necessary data to construct a consistent picture of the company's current situation regarding this topic, as well as its significant overlap with aspects already covered under the Knowledge Management domain.

To ensure reliability and validity, each interview was conducted by two researchers. Following the interviews, the researchers engaged in a thorough discussion and analysis to consolidate their findings. For the blue-collar, the obtained information was also reviewed and analyzed by the HR specialist, who managed relations with unions, and the foreman, who provided operational insights. Meanwhile, for the white-collar, the analysis was discussed with HR specialists and managers to ensure that the data reflected the perspectives and needs of each distinct group. These findings were then presented to the project manager and the designated company representative for validation. This collaborative approach ensured that the data were both comprehensive and aligned with the practical realities of the organization.

**Figure 3.**
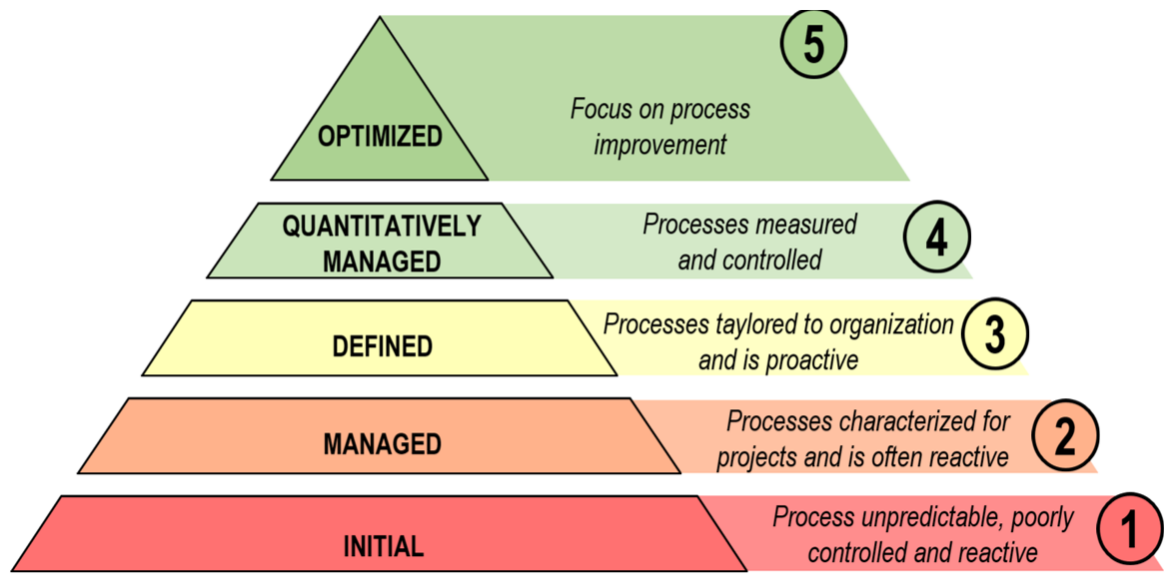
The maturity level ( Khraiwesh, 2020).

After analysing the responses for each domain, we develop a roadmap that incorporates the suggested actions and tools, as well as those already applied by the company ( Figure 5). The suggested action for each domain is derived from the MAIA framework and supported by the new ISO 25550:2022. This ISO standard aims to help organizations develop, implement, maintain and support an age-inclusive workforce ( ISO 25550: 2022). These actions are evaluated using the maturity model proposed by Khraiwesh (2020) (depicted in Figure 3), which categorizes the company’s readiness and progress across five levels, from 'Initial' to 'Optimized'. This model helps the company track its evolution, prioritize improvements, and implement continuous enhancements. By aligning each domain's actions with its maturity level, the company can strategically advance from reactive solutions to proactive, sustainable practices, fostering an inclusive, age-friendly work environment.

All detailed questions and their corresponding results are provided in Section 4. The results of the interviews were analysed within the context of the MAIA framework, existing literature, and ISO, ultimately leading to the development of a tailored roadmap for the company under study ( Figure 5).

## 4. Findings

Combining qualitative interviews with ergonomic assessments enabled a comprehensive understanding of the company's current aging worker initiatives and identified areas that require further development to create a more inclusive work environment for an aging workforce. The MAIA framework was deployed as a structural guide in data collection and analysis. The recommendations were based on the framework as well as related ISO 25550:2022

The company's readiness to address the challenges posed by an aging workforce was analyzed across five key domains of the MAIA roadmap: Organizational Culture and Leadership, Work Design and Ergonomics, Health Management, Knowledge Management, and Retirement Pathways. This section presents the findings for each domain, outlining the current issues and suggesting actions based on interviews, ergonomic assessments, and other data collection methods. In the following sections, we delve into each domain, discussing the company's priorities and highlighting areas that are either less considered or overlooked, based on the assessed maturity level.

### 4.1. Domain 1: Organizational culture and leadership

This domain focuses on the company’s culture and leadership practices, particularly their impact on aging workers. Unstructured questions were posed to 24 heads of functions responsible for both blue-collar and white-collar roles. The analysis of interviews with 24 heads of functions highlighted several issues related to aging workers within the company, focusing on job fit, hiring practices, training opportunities, and retirement perspectives (See in Table 4). Current challenges include bias in hiring aging workers, lack of processes for assessing job fit, limited opportunities for continuous learning, insufficient knowledge sharing, reluctance to engage in job rotation, and taboo about retirement. This taboo is more evident among white-collar employees, whose roles are less physically demanding. Unlike blue-collar workers, who often express a willingness to retire, white-collar employees may hesitate to openly discuss their desire for early retirement. Furthermore, workers’ hesitation to participate in job rotation stems from several underlying factors. Some employees lack confidence in their abilities and feel uncomfortable taking on new tasks due to concerns about making mistakes or the effort required for learning new processes and new technologies. These concerns can lead to a sense of stagnation and hinder organizational flexibility.

**Table 4.** The un-structured questions, current issues and suggested actions for Domain 1.

Interview sample Interviews with 24 heads of functions in charge.			
Collars (Blue & White)	Unstructured questions	Current issues	Suggested actions
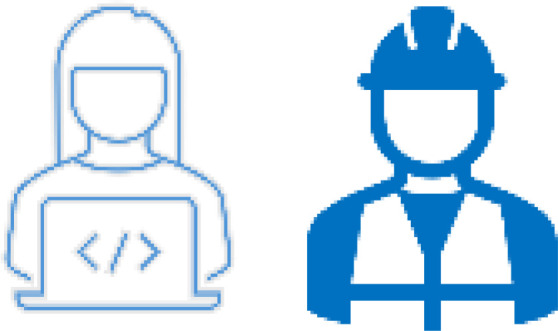	- Has the company recently hired or rehired ageing workers? How many and for what roles?	• Bias in hiring ageing workers 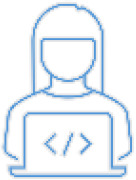	• Workshops and awareness program
	- Does your company have a process to assess job fit, especially for older employees?	• There is no process to assess job fit 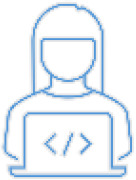 • Lack of listening to worker needs 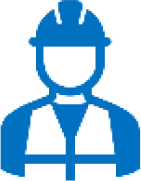	• Job assessment plans (job fit) • Raise workers awareness about their abilities
	- Do older employees have opportunities for continuous learning, ongoing training and skill development?	• Lack of continuous learning 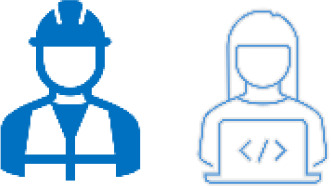 • Lack of knowledge sharing 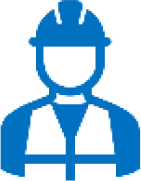	• Continuous learning opportunities • Recognition programs
	- How do you envision your ideal retirement lifestyle?	• Taboo about retiring 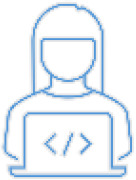 • Most workers are looking forward to retiring 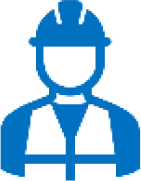	• Retirement Awareness Initiatives

Based on MAIA framework guidelines and our analysis of ground realities, we suggest the company consider a multi-pronged approach:

*Workshops and Awareness Programs:* Organize workshops to reduce age-related biases in hiring practices and to raise awareness among workers about their capabilities and strengths.

*Job Assessment Plans*: Establish processes for job fit assessment using appropriate tools, ensuring worker participation and input.

*Continuous Learning Opportunities*: Design continuous learning paths, including skill development programs tailored to older employees, to foster a growth mindset.

*Recognition Programs*: Implement reward-based systems to acknowledge the contributions of aging workers, thereby boosting motivation and engagement.

*Retirement Awareness Initiatives*: Facilitate open discussions about retirement to break the taboo and create plans that balance workers' aspirations and organizational needs.

### 4.2. Domain 2: Work design and ergonomics

This domain considers both cognitive and physical ergonomics. Hence, this domain evaluates the physical and mental workload associated with aging workers’ tasks using a semi-structured questionnaire based on the simplified NASA-TLX framework ( Noyes & Bruneau, 2007) ( Table 5). The simplified NASA-TLX questionnaire, with scores ranging from 1 (low workload) to 5 (high workload), was conducted with 52 blue-collar workers and foremen aged 55 and above to assess perceived physical and mental pressure. Based on these assessments, key issues were identified, as outlined in Table 5. These findings underline the dual challenges of physical strain and cognitive load faced by aging workers. Due to the subjective nature of NASA-TLX, we supplemented the analysis with RULA and REBA evaluations ( Ansari & Sheikh, 2014) for a subset of tasks that scored highest in the NASA-TLX questionnaire (e.g., one task in the assembly line). This task was perceived as highly physically demanding, and the results confirmed ergonomic concerns, with RULA scoring 6 and REBA scoring 5.

**Table 5.** The semi-structured questions, current issues, and suggested actions for Domain 2.

Interview sample Interviews with 52 workers/foremen aged 55 and older			
Collars (Blue & White)	Semi-structured questions	Current issues	Suggested actions by ISO
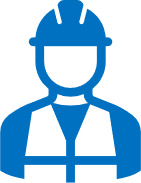	- How much physical activity is required? (e.g., pushing, pulling, lifting, turning) - How much physical pain or stress do you feel during or after task performance? (e.g., the amount of pain or pressure felt in areas such as the arm or back).	• High physical stress • Low worker's awareness about ergonomics importance while doing the tasks	• Worker’s involvement in job assessment • Role of mentor/trainer • Assistive technology • Postural training courses to increase ergonomic awareness
	- How much mental stress do you experience when performing tasks? - (e.g., cognitive demands like thinking, deciding, solving problems, precision, meeting deadlines, calculating, or remembering tasks)?	• High mental stress	• Worker’s involvement in job assessment • Role of mentor/trainer • Assistive technology
	- Does the company have a policy or approach for rotating tasks among workers?	• Poor job rotation • Temporary workers rotate and are trained, while permanent ones have fixed positions	• Planning job rotation

**Figure 4.**
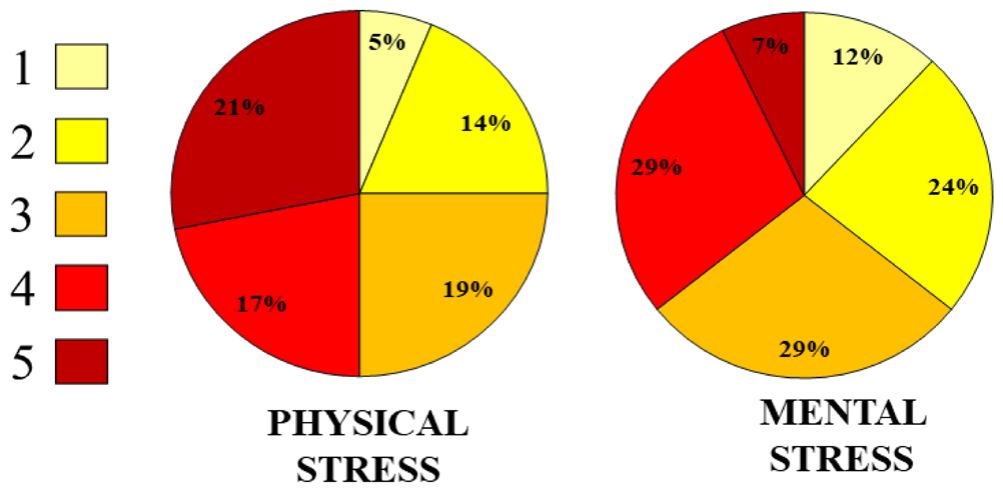
Percentage of perceived physical and cognitive workload among 52 aging Blue-Collar workers based on the simplified NASA-TLX questionnaire.

Furthermore, as shown in Figure 4, 38% of the aging blue-collar workforce reported experiencing a high physical workload (scores 4 and 5 on the NASA-TLX scale). Among them, 21% rated their physical workload at the highest level (score 5). The questionnaire was also applied again to specifically assess mental stress levels. According to these results, 36% of the respondents reported high levels of cognitive and mental stress (scores 4 and 5), highlighting the dual burden of physical and mental workload faced by aging workers.

These findings emphasize the need for targeted measures to address both physical and mental workload challenges. Additionally, based on the questionnaire results and our observations, it became clear that many workers were hesitant to take on new tasks. Their reluctance was largely driven by a mental barrier, rooted in the fear of learning new skills and the absence of a dedicated mentor or trainer to guide them through the process.

Hence, to address these issues, key interventions are proposed. First, increasing worker involvement in job assessments can help identify optimal job-fit solutions and empower employees to address challenges collaboratively. Second, introducing a trainer or mentor role within the workforce could alleviate both physical and mental stress. Aging workers with physical limitations could transition into mentorship roles, guiding younger colleagues while being partially relieved from physically demanding tasks. This mentorship strategy could address barriers such as reluctance to adopt new tasks, foster trust and comfort, especially if mentors are selected from the aging workforce, aligning with shared experiences ( Katiraee *et al.*, 2021b; Padula *et al.*, 2017). Third, leveraging assistive technologies, such as exoskeletons, weight-neutralizing devices, and collaborative robots (cobots), can reduce physical strain while enhancing productivity.

The company’s current ergonomic analysis tools, such as OCRA and NIOSH, used by the Environmental Health and Safety department, provide a foundation for further detailed assessments of assembly tasks. By building on existing methods and integrating new tools, such as RULA and REBA, a more comprehensive risk mitigation strategy can be implemented. These measures, alongside targeted job rotation planning and postural training programs, are essential for creating a safer and more supportive environment for aging workers.

### 4.3 Domain 3: Health management

Table 6 summarizes the unstructured questions, current issues, and suggested actions related to workers' health and well-being in the studied company. Interviews were conducted with personnel from both the blue and white-collar groups, including employees from the Personnel Administration and the Director of the Environment, Health, and Safety (EHS) department.

**Table 6.** The un-structured questions current issues and suggested actions for Domain 3.

Interview sample EHS members			
Collars (Blue & White)	Un-structured questions	Current issues	Suggested actions
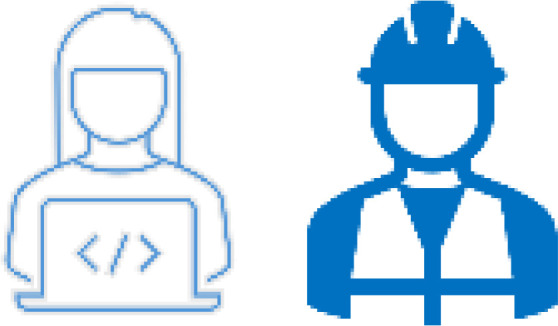	- What are the aspects that need improvements in the working environment?	• High physical and mental stress • Worker’s physical limitations • Lack of certain equipment (e.g. air conditioning at the production site) 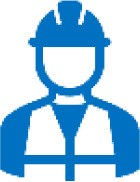	• Planning job rotation • Health extra screening • Improve the environment working conditions
	- Which are the active initiatives to take care about workers health and wellbeing?	• Physical inactivity after work 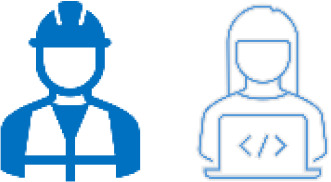	• Health promotion programs and activities to encourage workers to take care of their health

Key issues identified include high physical and mental stress, workers' physical limitations, and a lack of certain equipment at the production site. In response, the suggested actions involve planning job rotation, implementing extra health screenings, and improving working conditions— such as allowing time off for medical appointments, offering workplace sport or wellness programs, and providing more frequent breaks.

Additionally, addressing physical inactivity and promoting health and wellness programs are vital initiatives to encourage workers to focus on their health and well-being. These actions aim to create a healthier work environment and prevent long-term health issues among aging workers.

### 4.4 Domain 4: Knowledge management

Knowledge transfer is crucial, especially as many older workers hold key roles in R&D and operations. To ensure the continuity of specialized expertise, the company can introduce mentoring programs and leverage advanced tools like augmented reality (AR) to support intergenerational knowledge sharing. To identify critical roles within the company, interviews were conducted with 24 heads of functions responsible for both white- and blue-collar employees (see Table 2). These individuals were chosen for their comprehensive understanding of the essential skills and knowledge within their respective departments. The interviews began with two central questions aimed at assessing the importance and vulnerability of certain roles, as shown in Table 7.

**Table 7.** The unstructured questions current issues and suggested actions for Domain 4.

Interview sample Interviews with 24 heads of functions in charge.			
Collars (Blue & White)	Un-structured questions	Current issues	Suggested actions
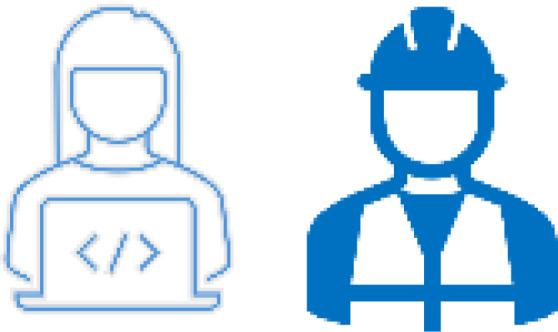	- What types of knowledge could be lost if someone retires? - How easy is it to replace a qualified person with this specific knowledge?	• White collar knowledge can be lost due to the lack of knowledge transfer • No tool to see the critical roles • Due to the lack of knowledge transfer it is difficult to replace a qualified person 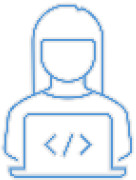 • Poor knowledge mapping (operative issues about time, not attitude) 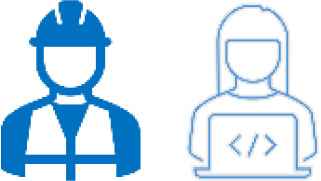	• Role of mentor/trainer • Intergenerational meetings • Reward based program • Enhance mapping methods about knowledge

The interviews revealed a deeper understanding of roles classified as critical roles that possess highly specialized knowledge and skills difficult to replace in the labor market. A role is deemed critical when its expertise is concentrated in a small number of individuals, and the knowledge is not easily transferable or widely available. A major risk arises when workers nearing retirement do not fully transfer their knowledge to the next generation, underscoring the need for succession planning well before retirement. Mapping the skills of both younger and older employees is vital, along with implementing an appropriate succession plan to facilitate a smooth transition of knowledge.

### 4.5 Domain 6: Retirement pathways

The investigation into the "Retirement Pathways" domain focuses on both the technical and cultural aspects of an employee's career end, particularly for both blue-collar and white-collar workers ( Table 8). To gather relevant information, interviews were conducted with key personnel, including the head of personnel administration for both collar types and the human resource business partner, especially for blue-collar workers, due to their close interaction with labour unions. Additionally, during the interviews for Domain 4 on "Knowledge Management," valuable insights into retirement pathways also emerged.

**Table 8.** The unstructured questions, current issues, and suggested actions for Domain 6.

Interview sample With personal administration			
Collars (Blue & White)	Unstructured questions	Current issues	Suggested actions by ISO
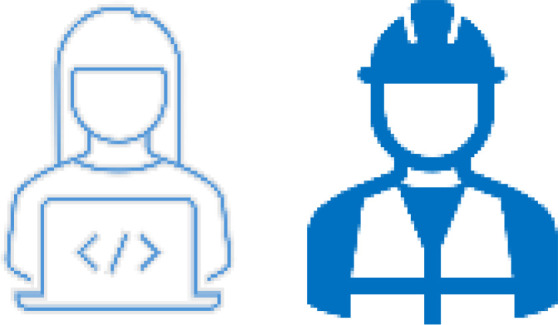	- At the time the worker is going into retirement, is it the company that asks him his retirement intentions or the does the worker himself communicate retirement intentions or plans?	• Workers want to retire 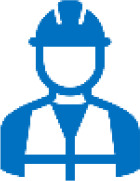 • Taboo about retiring 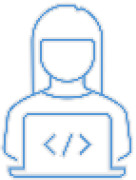	• Flexible time arrangements and work allocation • Reward-based program
	- Is there a succession plan?	• Mentoring is not planned 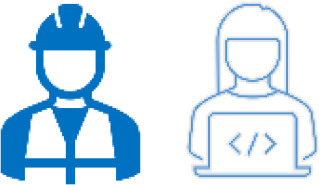	• Succession plan

One of the most significant issues identified is the absence of a systematic succession plan that facilitates smooth transitions of knowledge and skills between generations. Currently, succession planning exists only in isolated, high-level management cases, but there is no formal, systematic approach for general workforce roles. A comprehensive succession plan should be established, starting well before a worker's retirement. This plan should involve the aging worker, serving as a mentor, to ensure the transfer of know-how to younger colleagues, thereby ensuring continuity within the workforce. Furthermore, the company should develop flexible retirement pathways to accommodate the aging workforce, offering options such as gradual retirement and retraining programs. This will enable older employees to transition out of the workforce smoothly while continuing to contribute in meaningful ways during this process.

## 5. Developed roadmap and discussion

**Figure 5.**
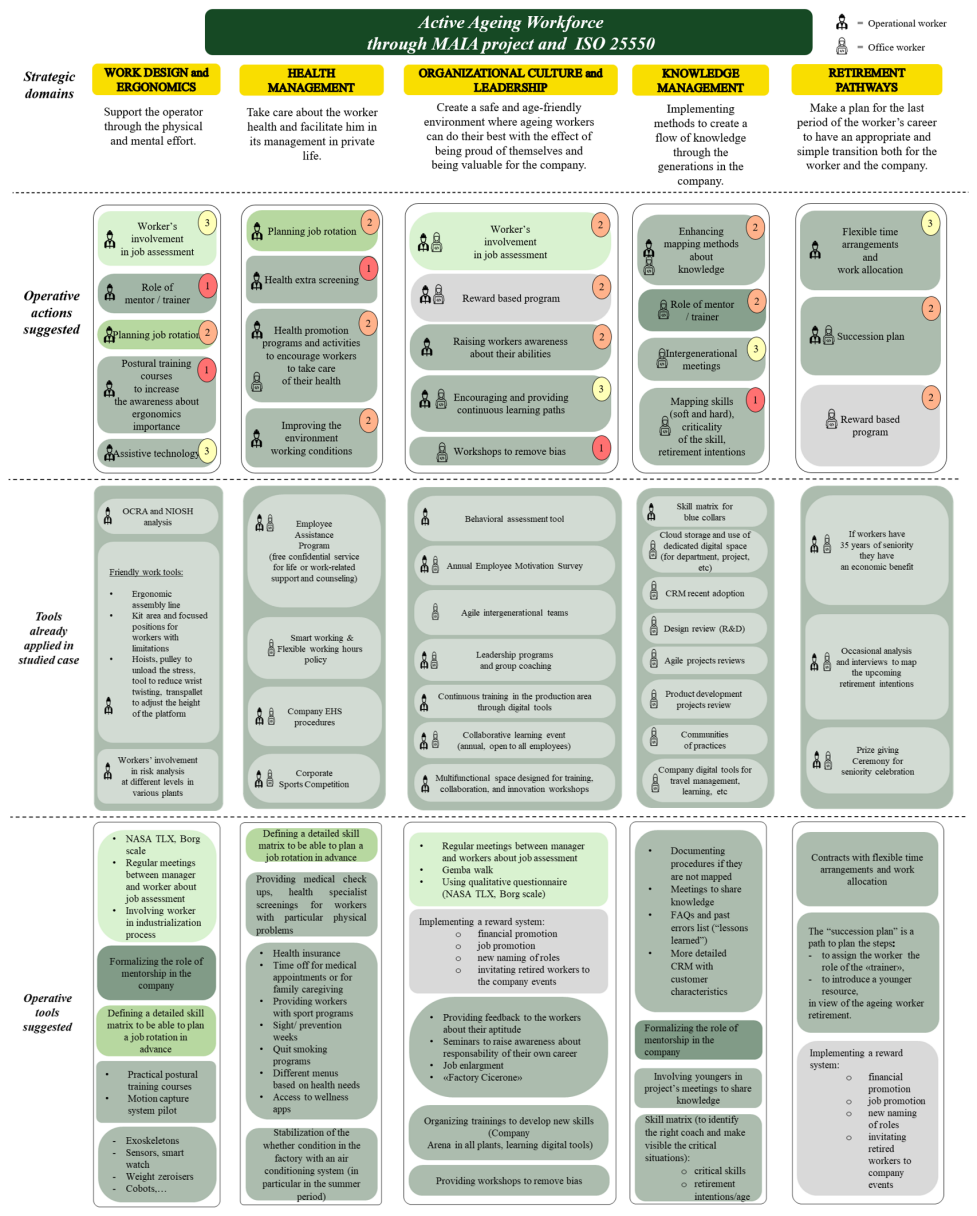
Roadmap about active ageing workforce in the studied company by strategic domains, operative actions suggested, tools already applied in the company, and operative tools suggested.

Based on the findings presented in Section 4, we developed the final roadmap for managing the aging workforce, as illustrated in Figure 5. This roadmap outlines the strategic domains, operative actions, and tools currently in use within the company. The roadmap addresses the challenges associated with an aging workforce through targeted actions across five key domains: Work Design and Ergonomics, Health Management, Organizational Culture and Leadership, Knowledge Management, and Retirement Pathways. Each domain focuses on enhancing workplace conditions, supporting employees' health, promoting inclusive leadership, facilitating knowledge transfer, and ensuring smooth transitions into retirement. As mentioned earlier, the roadmap is evaluated through a maturity model, depicted in Figure 3, which categorizes the company's readiness and progress into five levels, ranging from 'Initial' to 'Optimized.' The assessment was conducted using a set of predefined criteria across key domains such as organizational culture, workplace design, and HR practices. Data sources included interviews with the head of the functions and employees, site observations, and internal policy reviews.

This model enables the company to track its evolution, prioritize improvements, and implement continuous enhancements. By aligning each domain's actions with its maturity level, the company can strategically progress from reactive solutions to proactive and sustainable practices that foster an inclusive, age-friendly work environment.

The company has already implemented a range of tools to support the aging workforce across different domains. In the area of Work Design and Ergonomics, tools such as the ergonomic assembly line, hoists, and adjustable platforms have been put in place to reduce physical strain and support workers with limitations. Additionally, the company uses health-related tools, including the ‘Smart Working’ system, time flexibility options, and the 'EHS procedures,' to promote better work-life balance and worker well-being. For knowledge management, the company utilizes a skill matrix for blue-collar workers, along with aptitude assessment tools like Saper, Super sense, and Super fare, which help identify the critical roles and map the skillsets of employees. Furthermore, in the domain of retirement pathways, the company offers economic benefits for workers with 35 years of seniority and conducts occasional interviews to understand workers' retirement intentions. These tools are essential in addressing the needs of an aging workforce, ensuring that the company is equipped to foster a supportive, inclusive, and efficient work environment.

Furthermore, the roadmap outlines a variety of suggested tools to enhance the company's ability to support an aging workforce across the five strategic domains. For *Work Design and Ergonomics*, tools like motion capture systems, exoskeletons, and smart sensors are proposed to mitigate physical strain, alongside practical postural training courses to raise ergonomic awareness ( Battini *et al.*, 2022). In the *Health Management* domain, advanced health checkups, screening programs, and specialized tools for stress management, such as meditation apps and tailored nutrition packages, are recommended to promote well-being. For *Organizational Culture and Leadership*, suggested tools include structured feedback mechanisms, seminars to enhance career responsibility, and innovative engagement approaches like gamified training sessions and workshops to combat bias. Within *Knowledge Management*, tools such as a skill matrix for identifying critical roles, digital CRM systems for marketing and sales, and platforms for documenting and sharing best practices (e.g., FAQs and lessons learned) are emphasized to support effective knowledge retention and transfer. Finally, under *Retirement Pathways*, the proposed tools include succession planning frameworks, flexible work arrangements, and reward systems that provide financial incentives or career development opportunities, ensuring a smooth transition for retiring employees while maintaining organizational continuity.

These tools are tailored to the unique needs of aging workers while aligning with the company’s operational goals, fostering a holistic and forward-looking approach to workforce management.

### 5.1 The Relevance of the MAIA framework in a real-world context

The findings of this study indicate that the MAIA framework provides a valuable structure for assessing the various dimensions of workforce aging. Its focus on five key domains—organizational culture and leadership, work design and ergonomics, health management, knowledge management, and retirement pathways—covers essential aspects for maintaining the productivity, well-being, and integration of aging employees in the workplace. In the studied company, each of these domains was found to be highly relevant, especially in a manufacturing setting where physical demands and knowledge transfer are critical.

For example, domain 2: work design and ergonomics proved to be particularly significant, as ergonomic risks were prevalent, especially in blue-collar positions such as assembly line workers. The application of ergonomic assessment tools like RULA and REBA highlighted the need for immediate improvements in job design and rotation programs to reduce physical strain. Similarly, in domain 4: knowledge management, the study revealed a strong reliance on older workers for critical tasks, particularly in R&D and operations, underscoring the importance of formalizing knowledge transfer processes through mentoring and training.

The overall structure of the MAIA roadmap facilitated a systematic approach to identifying gaps in the company's existing policies and practices. However, despite the framework’s relevance, its practical application was not without challenges.

### 5.2 Challenges in implementing the MAIA framework

While the MAIA framework offers a robust roadmap, its implementation in a real-world setting revealed several challenges:

***5.2.1   Cultural resistance and organizational inertia***

The company showed a reluctance to fully engage with certain domains of the MAIA framework, particularly domain 1. There was a lack of awareness and active involvement from senior leadership in discussions around the aging workforce, leading to inadequate attention to job fit and retention strategies for older workers. Cultural resistance to acknowledging aging as an integral workforce issue can significantly hinder progress. Despite workshops being proposed to address biases and age-related stigmas, there was limited enthusiasm for such initiatives.

***5.2.2   Lack of trainers and mentors to support older workers***

A critical gap was identified in the absence of dedicated trainers and mentors, particularly those who could be aging workers themselves, to guide others in learning new tasks. This was particularly evident in domains 2 and 4. The reluctance of many aging workers to take on new roles was not only due to physical strain but also rooted in mental barriers, such as fear of learning new skills. The lack of experienced mentors—especially those with similar age or experience—contributed to longer learning times and heightened anxiety. Introducing aging mentors could help reduce these barriers and speed up the learning process by providing relatable support.

***5.2.3  Knowledge transfer and succession planning***

Domain 4 highlighted another significant challenge—knowledge transfer. Despite the critical roles older workers play in areas like R&D, the company had no formalized knowledge transfer systems or mentoring programs in place. While the company recognized the risks of knowledge loss, especially as experienced workers approach retirement, there was no established mechanism to systematically capture and transfer this knowledge to younger workers. This issue was further compounded by the absence of succession planning in domain 6, where there was a clear need for more structured retirement and mentoring processes to ensure a smoother transition.

### 5.3 Practical implications and suggested improvements

Despite the challenges, the study highlights several actionable areas where the company could improve its management of an aging workforce:

***5.2.4  Workshops and training for leadership and HR***

To overcome cultural resistance, leadership workshops and targeted HR training are essential. These initiatives should focus on reducing age-related bias and promoting a more inclusive mindset, where aging is viewed as an asset rather than a liability. These efforts could significantly improve job fit assessments, worker retention, and job satisfaction among older employees.

***5.2.5  Investment in ergonomic solutions and health programs***

While budgetary constraints may limit immediate, large-scale ergonomic improvements, the company could start with smaller, high-impact interventions, such as adjustable workstations and targeted postural training. Additionally, encouraging physical activity and promoting health through low-cost initiatives, such as fitness programs or stress reduction workshops, could improve workers' physical well-being without requiring substantial financial investment.

***5.2.6  Formalizing knowledge transfer and mentoring programs***

Establishing structured mentoring and knowledge transfer programs is essential for mitigating the risks of knowledge loss. This could involve pairing older workers with younger employees in formal mentorship roles, as well as leveraging digital tools, such as augmented reality (AR), to facilitate knowledge sharing. Additionally, the development of knowledge-mapping tools to identify critical roles and expertise within the company would ensure that vital skills are not lost when older workers retire.

***5.2.7  Flexible retirement pathways and succession planning***

Gradual retirement pathways, including options for phased retirement and flexible work arrangements, would allow older workers to transition out of the workforce at a pace that benefits both them and the company. Developing succession plans in advance, coupled with mentoring programs, would also ensure continuity and smooth transitions for critical roles.

### 5.4 Broader implications for manufacturing industries

The findings of this case study offer broader insights for other companies within the manufacturing sector facing similar challenges. The MAIA framework, while comprehensive, needs to be adapted to each company's unique context, balancing resource constraints with strategic priorities. Manufacturing industries, in particular, need to consider the high physical demands of many roles and invest in ergonomic solutions and health management programs to safeguard their aging workforce. Additionally, fostering an organizational culture that values intergenerational collaboration and knowledge transfer is critical for sustaining long-term productivity and innovation.

## 6. Conclusion

This research aimed to investigate aging workforce issues in a manufacturing setting, applying the MAIA framework. The new ISO: 25550 standards on aging societies were also consulted. The study began by assessing critical issues across each MAIA framework domain through information gathered from operational practices, tools present in the company, and insights into the company’s working culture. This included informal behaviours and attitudes among employees that influence the workplace but are not formally documented.

Once the company’s current challenges were identified, the MAIA framework and ISO: 25550 guidelines helped prioritize urgent actions, and appropriate tools were recommended to foster an active aging workforce. The result was a customized roadmap, designed to guide the company in implementing age-friendly policies across various departments, ensuring that the suggested actions and tools are applied to meet the organization's needs.

One key finding was the central role of the "Organizational Culture and Leadership" domain, which emerged as a foundational pillar for implementing age-friendly strategies. Organizational culture shapes team cohesion, workplace climate, and overall effectiveness, making it a crucial starting point for fostering age-inclusive policies that enable the success of other strategic domains. Two other domains that held particular importance were "Work Design and Ergonomics" and "Knowledge Management".

Ergonomics took center stage for blue-collar workers. The NASA-TLX questionnaire and ergonomic assessments, including RULA and REBA, revealed the need for greater job rotation and individualized job assignments, essential for promoting health and well-being in the workplace.

"Knowledge Management" emerged as critical issue for white-collar employees, particularly in preserving the expertise and competencies of aging workers. Formal knowledge mapping processes and the creation of intergenerational knowledge transfer mechanisms, notably through the introduction of "trainer/mentor" roles were recommended remedies.

The final roadmap revealed a synergistic effect across all domains, with certain actions—such as implementing job rotation and creating trainer/mentor roles—proving beneficial across multiple areas of the organization. This highlights not only the interconnection between different domains but also the substantial improvement potential that can arise from even a few targeted actions. The developed roadmap is thus instrumental in helping the company strategically navigate workforce aging, offering practical steps for improving productivity, worker well-being, and intergenerational collaboration.

### 6.1 Study limitations

There were certain limitations encountered in the study. It was not possible to suggest comprehensive actions for the "Intergenerational Coexistence" domain of the MAIA framework due to difficulties in collecting consistent information across the company's various departments. This topic requires further investigation to gain a complete understanding of the current situation in this dimension of the workplace environment.

In the "Work Design and Ergonomics" domain, while ergonomic risks were assessed using RULA and REBA, advanced technologies like motion capture systems could be employed in future research to provide more precise results and analyze more workstations. Additionally, involving a larger sample of workers in ergonomic assessments, considering factors such as gender, age, and body type, would offer a more comprehensive analysis.

Considering all domains, the study could be extended to implement the proposed tools and evaluate their effectiveness and adaptability in the workplace.

Overall, the MAIA framework should be applied to a broader range of industrial companies, allowing for the development of roadmaps tailored to specific needs. This would support businesses in addressing the demographic changes affecting today's workforce. The expansion of these actions would help companies build resilience in response to future workforce shortages. Further research is needed to deeply investigate the implications of this trend and how it will affect the future of organizations.

## Ethics and consent

Formal ethical approval was not obtained from an Institutional Review Board (IRB), as the study was conducted internally with organizational approval from the participating company. The company reviewed and approved the research procedures before the start of the study. At the time of the research, the study was assessed to involve no risk and did not include sensitive or medical personal data requiring IRB oversight. All research procedures complied with the principles of the Declaration of Helsinki.

All participants provided written informed consent before taking part in the study. They were informed about the purpose of the research, the voluntary nature of their participation, their right to withdraw at any time, and the confidentiality of their responses

## Data consent

The data generated and/or analyzed during the current study are not publicly available due to confidentiality agreements with the participating company and the difficulty in fully anonymizing the qualitative interview data. However, access to redacted data may be granted on reasonable request. Interested researchers should contact the corresponding author at [ niloofar.katiraee@unipd.it], outlining the purpose of their request. Access may be granted at the discretion of the authors and by ethical and legal considerations.

## Data Availability

The interview data generated during the current study are not publicly available due to confidentiality agreements with the participating company and the nature of the organizational case study. The data contains context-specific insights that cannot be fully anonymized without compromising participant privacy and organizational confidentiality. However, access to redacted data may be granted upon reasonable request. Interested researchers may contact the corresponding author at **niloofar.katiraee@unipd.it**, outlining the purpose of their request. Access will be considered at the discretion of the authors and subject to ethical and legal requirements.
