# Supplementary material for: A roadmap for managing an ageing workforce in the manufacturing sector: An Italian case study
Source: Open Res Eur. 2025 Aug 26;5:253. [Version 1] doi: 10.12688/openreseurope.20511.1 (PMC12444273; doi:10.12688/openreseurope.20511.1)
Supplement: Peer review history

# Reviewer report: Reviewer response for version 1

**DOI:** 10.21956/openreseurope.22195.r59238

**Published:** 2025-10-06

**Authors:** Anna Corinna cagliano

**Affiliations:** 1 Politecnico di Torino, Torino, Italy

The manuscript presents a case study on workforce management and ergonomics in a manufacturing company, focusing on aging employees. It provides a clear overview of the company’s current characteristics, including sector, size, global presence, workforce composition, and adoption of Agile practices. The study applies the MAIA framework and ISO 25550:2022 standards, using descriptive ergonomic assessments (NASA-TLX, RULA, REBA) to evaluate working conditions. The manuscript is well-structured, supported by informative figures and tables, and includes a plain language summary that enhances readability. Overall, it offers practical insights relevant to both researchers and practitioners in workforce sustainability and ergonomics.

 Please, find my detailed comments below:

1. All underlying data are provided, ensuring full reproducibility. 
2. The conclusions are only partially supported by the results. While the study presents valuable observations, the manuscript would benefit from explicitly linking each recommendation to the evidence provided. For example, clarifying how the ergonomic assessments directly informed the proposed roadmap for aging workforce management would strengthen the scientific rigor.
3. The case is described in sufficient detail to be valuable for practitioners and educators, effectively illustrating the practical implementation of workforce management strategies in a manufacturing setting.
4. The current characteristics of the company are clearly described, but the historical development of workforce demographics, HR strategies, and organizational challenges is only partially addressed. Including a chronological perspective on trends in workforce composition, past HR policies, and challenges in knowledge transfer, ergonomics, or retirement planning would provide a more complete understanding of the case’s evolution and enrich the context for the roadmap presented.
5. The manuscript is clearly written, with a logical structure and supportive visuals. However, the literature review relies heavily on the authors’ prior work. Incorporating a broader range of contemporary studies on aging workforce management, ergonomics, and HR practices in manufacturing would situate the case more firmly within the academic debate and provide stronger external validation of the applied framework.
6. The study employs a qualitative case study approach complemented by descriptive ergonomic assessments, which is appropriate for the research objectives. No inferential statistics were applied.

To enhance the manuscript’s scientific rigor, I suggest the authors to:

- Strengthen the connection between results and conclusions, explicitly showing how each recommendation is supported by the evidence.
- Expand the historical context of workforce demographics, past HR strategies, and organizational challenges to provide a richer understanding of the case’s progression.
- Broaden the literature review to include independent, recent studies on aging workforce management and ergonomics, reducing reliance on self-citations and situating the work within the wider research landscape.

Good luck!

Is the case presented with sufficient detail to be useful for teaching or other practitioners?

Partly

Is the work clearly and accurately presented and does it cite the current literature?

Partly

If applicable, is the statistical analysis and its interpretation appropriate?

Not applicable

Are all the source data underlying the results available to ensure full reproducibility?

Yes

Are the conclusions drawn adequately supported by the results?

Yes

Is the background of the case’s history and progression described in sufficient detail?

Partly

Reviewer Expertise:

Design of industrial facilities, industrial logistics

I confirm that I have read this submission and believe that I have an appropriate level of expertise to confirm that it is of an acceptable scientific standard, however I have significant reservations, as outlined above.

# Reviewer report: Reviewer response for version 1

**DOI:** 10.21956/openreseurope.22195.r59240

**Published:** 2025-09-17

**Authors:** Lisa Meehan

**Affiliations:** 1 Auckland University of Technology (AUT), Auckland, New Zealand

This is a thoughtful and practice-oriented paper on a pressing topic. The authors apply the MAIA framework and ISO 25550:2022 to a real-world manufacturing case in Northern Italy. The study draws on interviews with older blue- and white-collar employees, input from managers and HR, and ergonomic assessments (NASA-TLX, RULA, REBA). The resulting roadmap is both timely and relevant, and the mixed-methods approach is a particular strength.

**Major comments**

 1. Clarify the main contribution: The paper would benefit from stating more clearly whether its central strength lies in the empirical case study, the conceptual use of MAIA, the practical roadmap, or in the combination of these elements.

2. Intergenerational coexistence domain: Domain 5 was omitted, yet it is central to knowledge transfer. Even if data were limited, the paper could suggest how this might be explored in the future (e.g. through workshops or targeted surveys). 

3. Roadmap and maturity model: The roadmap is useful, but the maturity model results are not fully shown. A table or chart indicating the company's current level in each domain could be helpful. 

4. Context and transferability *:* **The discussion could more explicitly distinguish between what is specific to Northern Italy (e.g. union structures, demographics) and what is more generally applicable. This would help readers gauge the wider relevance of the roadmap. 

5. Succession planning and knowledge transfer: The paper identifies these as issues but if it is feasible, it could point to concrete tools (e.g. digital knowledge repositories or succession plan templates) to give practitioners more practical guidance. 

**Minor suggestions**

 - Use "ageing" or "aging" consistently

 - Consider simplifying some longer sentences for readability

 - Consider replacing the term *foremen* with a more gender-neutral alternative, such as *supervisor* or *shift supervisor*

Is the case presented with sufficient detail to be useful for teaching or other practitioners?

Yes

Is the work clearly and accurately presented and does it cite the current literature?

Yes

If applicable, is the statistical analysis and its interpretation appropriate?

Yes

Are all the source data underlying the results available to ensure full reproducibility?

Yes

Are the conclusions drawn adequately supported by the results?

Yes

Is the background of the case’s history and progression described in sufficient detail?

Yes

Reviewer Expertise:

Economics

I confirm that I have read this submission and believe that I have an appropriate level of expertise to confirm that it is of an acceptable scientific standard.

# Reviewer report: Reviewer response for version 1

**DOI:** 10.21956/openreseurope.22195.r59555

**Published:** 2025-09-09

**Authors:** Antonella Petrillo

**Affiliations:** 1 University of Naples “Parthenope”, Napoli, Italy

The article "A roadmap for managing an aging workforce in the manufacturing sector: An Italian case study" presents an analysis of the challenges associated with managing an aging workforce in a multinational manufacturing company in Northern Italy. The authors apply the MAIA (Models and Methods for an Active Aging Workforce) framework, integrated with the ISO 25550:2022 standard, to assess five key domains: organizational culture and leadership, ergonomics and work design, health management, knowledge management, and retirement pathways.

 Through interviews (103 participants), field observations, and ergonomic assessments (NASA-TLX, RULA, REBA), the authors develop a roadmap of targeted actions that includes tools already implemented and future proposals. The results highlight strengths (use of ergonomic tools, organizational flexibility, skill matrix) and weaknesses (lack of succession planning, cultural resistance, lack of structured mentoring programs). The main contribution is the practical demonstration of how the MAIA framework can guide age-inclusive policies in the manufacturing sector.

 The paper addresses a real and urgent challenge, with strong practical and policy interest. However, the article presents some critical issues and weaknesses, as detailed below.

 1. The "Intergenerational coexistence" domain has been omitted. Add details. Clarify and propose a methodology for the missing domain.

 2. It is stated that it was not possible to collect consistent data. However, intergenerationality is central to knowledge transmission. Clarify more systematically why data were not available and propose a future methodology (e.g., mixed young/old focus groups, comparative survey) to fill this gap.

 3. The study is based on a single case study, which limits external validity. Include a more detailed section on methodological limitations and indicate how the results could be verified in other contexts (e.g., comparison with manufacturing SMEs or companies in other sectors).

 4. Succession planning and knowledge transfer. Although identified as a critical issue, a concrete proposal for digital or process-based tools (beyond AR and mentoring) is lacking. Provide practical examples of already validated systems (e.g., knowledge management platforms, skills databases) and assess their applicability to the company studied.

 5. RULA/REBA evaluations were conducted only on a limited subset of activities. Further specify the selection criteria and discuss the methodological implications (possible bias). Recommend future studies with a larger sample size and the use of advanced technologies (e.g., motion capture).

 6. The article acknowledges cultural resistance but does not delve into how this influences industrial relations (e.g., the role of unions). Expand the discussion to include union dynamics or Italian regulations, which could impact phasing-out processes or job rotation.

 7. Formal IRB approval is stated to be lacking. While not mandatory in a corporate context, it would be helpful to specify what specific privacy protection measures were applied (beyond written consent).

 8. Strengthen the discussion on the generalizability of the findings beyond the individual company case.

 9. Further detail the sampling and criteria in ergonomic analyses.

 10. Specify how cultural and union resistance can hinder/facilitate the implementation of the roadmap.

 The article provides an important and original contribution to the application of the MAIA framework in a real-world context. With tminor revision the study will become an even more solid and useful reference for both the scientific community and companies dealing with the challenges of an aging workforce.

Is the case presented with sufficient detail to be useful for teaching or other practitioners?

Yes

Is the work clearly and accurately presented and does it cite the current literature?

Yes

If applicable, is the statistical analysis and its interpretation appropriate?

Yes

Are all the source data underlying the results available to ensure full reproducibility?

Yes

Are the conclusions drawn adequately supported by the results?

Yes

Is the background of the case’s history and progression described in sufficient detail?

Yes

Reviewer Expertise:

Industrial Plant

I confirm that I have read this submission and believe that I have an appropriate level of expertise to confirm that it is of an acceptable scientific standard.
